# Vitamin E promotes dermal papilla cell proliferation via the lncRNA2919/STAT1 axis to activate the Hedgehog signaling pathway

**DOI:** 10.1016/j.ncrna.2026.06.005

**Published:** 2026-08-18

**Authors:** Yinuo Fang, Sen Wang, Chengpeng Cao, Anqi Yang, Jing Zhang, Bohao Zhao, Xinsheng Wu, Yan Liu, Yang Chen

**Affiliations:** a College of Animal Science and Technology, Yangzhou University, Yangzhou, 225009, China; b Zhejiang Key Laboratory of Livestock and Poultry Biotech Breeding, Institute of Animal Husbandry and Veterinary Science, Zhejiang Academy of Agricultural Science, Hangzhou, 310021, China

**Keywords:** Vitamin E, Hair follicle, Dermal papilla cells, lncRNA2919, *STAT1*, Hedgehog signaling pathway

## Abstract

Hair-follicle development is a key determinant of the quality and yield of Angora rabbit wool. Vitamin E (VE) has antioxidant properties and regulates cell proliferation. However, the molecular mechanism by which VE regulates hair-follicle growth is unclear. This study investigated the effects of VE on hair follicles and dermal papilla cells (DPCs) *in vitro,* as well as their underlying regulatory mechanism involving long noncoding RNA (lncRNA)2919, *STAT1*, and the Hedgehog signaling pathway. Hair follicles and DPCs were isolated and cultured. The optimal VE concentration was determined to be 50 μM. Reverse-transcription quantitative PCR, Western blotting, and CCK-8, DNA pulldown, chromatin immunoprecipitation, and dual-luciferase reporter assays revealed that VE significantly promoted both hair-follicle growth and DPC proliferation, and also regulated the expression of hair follicle-development–related genes, such as *LEF1*, *FGF2*, *TGFβ1*, and *SFRP2*, and antioxidant genes, including *SOD1* and *NRF2*, and also enhanced the antioxidant capacity. Mechanistically, VE significantly downregulated lncRNA2919 expression, while lncRNA2919 overexpression inhibited DPC proliferation, which was reversed by VE. Furthermore, lncRNA2919 maintained STAT1 protein levels by inhibiting the proteasome-mediated degradation pathway. STAT1 was observed to bind directly to the SHH promoter and inhibit its transcription. This effect was reversed by VE via downregulation of lncRNA2919*/STAT1* and subsequent activation of the Hedgehog signaling pathway. In conclusion, VE induces the proliferation of DPCs and enhances cellular antioxidant capacity by regulating the lncRNA2919/*STAT1*/*SHH* axis, thereby promoting hair-follicle growth and development. The findings provide a novel theoretical basis for understanding hair-follicle growth and the use of VE in Angora rabbits.

## 1 Introduction

Hair-follicle development and hair growth are regulated by multiple factors and several signaling pathways [1,2]. There is evidence that oxidative stress contributes significantly to hair loss [3]. Vitamin E (VE) is a classic lipophilic antioxidant molecule that can eliminate lipid peroxides associated with skin and hair diseases [4] and interact directly with lipid radicals [5], thereby playing a unique role in the maintenance of skin and hair health [6]. The scalps of patients with hair loss typically show reduced levels of antioxidants, accompanied by a higher lipid peroxidation index [7,8]. Orally administered capsules of tocotrienol, a member of the VE family, can reduce lipid peroxidation levels and alleviate oxidative stress in the scalp [9]. VE promotes hair growth by enhancing both the antioxidant capacity and local blood circulation to optimize the hair-follicle microenvironment [10]. Dermal papilla cells (DPCs) are the regulatory center of hair-follicle development, and secrete various signaling molecules, such as transforming growth factor (TGF)-β, fibroblast growth factor (FGF), and Sonic Hedgehog (SHH), thereby regulating the hair-growth cycle and maintaining hair follicle homeostasis [11,12]. The number of DPCs is a key determinant of hair size, shape, and growth cycle [13]. However, to the best of our knowledge, there have been no investigations into the effect of VE on the proliferation of DPCs.

Long noncoding RNAs (lncRNAs) perform crucial biological functions and regulate multiple biological processes [[14], [15], [16]]. Specific lncRNAs, such as ANCR, TINCR, HOTAIR, and SPRY4-IT1, are involved in the biological regulation of the hair-follicle cycle [17]. Our previous whole-transcriptome sequencing of Angora rabbit hair follicles across different cycles revealed low expression of lncRNA2919 in the anagen phase and high expression in the catagen phase, suggesting that it may play a key role in hair-follicle growth [18]. It is known that VE promotes hair-follicle growth [10]. However, the involvement of lncRNA2919 in VE-regulated hair-follicle growth remains unclear.

The transcription factor STAT1 has been observed to regulate both the hair-growth cycle and follicle development [19]. STAT1 interacts with the Hedgehog (Hh) signaling pathway and is widely involved in various physiological and pathological processes, such as cell differentiation, immune regulation, and tumorigenesis [[20], [21], [22]]. Hedgehog signaling is crucial for hair-follicle development and maintenance of growth-cycle homeostasis [23]. The Hh signaling pathway includes three Hh ligands, Sonic Hedgehog (Shh), Indian Hedgehog (Ihh), and Desert Hedgehog (Dhh) [24]. The Shh pathway is a critical regulator of hair-follicle morphogenesis, cycling, and regeneration, and is essential for the telogen-to-anagen transition and for maintaining the proliferative capacity of hair-follicle stem cells [25]. RNA-binding protein immunoprecipitation assays to confirm that lncRNA2919 could target and regulate the expression of *STAT1* [26]. Based on prior studies and our previous findings, we therefore hypothesize that VE promotes Hh signaling by regulating signal transduction within the lncRNA2919/*STAT1* axis, thereby modulating hair-follicle growth in Angora rabbits.

The optimal concentration of VE was determined *in vitro* using hair follicles and DPCs from Angora rabbits. The regulatory effects of VE on DPC proliferation, as well as the influence of lncRNA2919 on STAT1 protein stability and subsequent signal transduction by the downstream Hedgehog signaling pathway, were investigated. The findings provide a novel theoretical basis and identify factors associated with the molecular network underlying hair-follicle growth in Angora rabbits.

## 2 Methods

### 2.1 Animals

All experimental procedures involving animals were approved by the Animal Care and Use Committee of Yangzhou University (Approval No: 202301018). The Angora rabbits used in the study were raised under the same controlled conditions. After euthanasia, vibrissa skin tissues were collected for subsequent experiments.

### 2.2 Isolation and *in vitro* culture of hair follicles

Two-month-old Angora rabbits were euthanized, and the vibrissa skin tissues were collected and placed in a 50-mL centrifuge tube containing D-Hank's solution (Solarbio, Beijing, China). After washing with PBS containing 5% penicillin-streptomycin, the tissues were immersed in 4% collagenase IV (Gibco, Waltham, MA, USA) and digested at 37°C for 1 h. Individual hair follicles were rewashed with D-Hank's solution. Individual hair follicles were placed in the wells of 24-well plates, and basal culture medium was prepared (Supplementary Table 1). A dimethylsulfoxide (DMSO) control group and four VE treatment groups (10 μM, 30 μM, 50 μM, 70 μM) were established based on preliminary experiments. Images were taken on days 0, 2, and 4 to observe hair-shaft growth.

### 2.3 Isolation and culture of primary DPCs

The dorsal skin of the rabbits was removed and placed in PBS, and the fat layer was removed. The skin samples were cut into square strips and placed in a culture dish with the dermis side up. The samples were treated with neutral protease (Gibco) and digested overnight at 4°C. The following day, the dermal layer was transferred to a new culture dish with 8 mL EDTA (Gibco). Digestion was halted after digestion for 30 min at 37°C. The culture supernatant was filtered and centrifuged at 360 ×g for 10 min, after which the supernatant was discarded, and mesenchymal stem cell medium (MSCM, ScienCell, Carlsbad, CA, USA) containing 1% penicillin-streptomycin (Solarbio) was added. The sample was mixed gently and incubated at 37°C. The medium was replaced after 12 h. Subculturing was performed when the cell density was sufficient.

### 2.4 Immunofluorescence staining

DPCs were seeded into 12-well plates. When 70–80% confluent, the cells were fixed with 4% paraformaldehyde (Solarbio), permeabilized with Triton X-100 (Solarbio), and blocked with a blocking solution (NcmBlot Blocking Buffer, New Saimei Biotech, Suzhou, China) for 10 min. Primary antibodies against α-SMA (Boster, Wuhan, China, 1:200) and vimentin (Boster, 1:200) were added. After incubating overnight at 4°C, the cells were washed with PBS. Cy3-labeled goat anti-mouse secondary antibody solution (Proteintech, Wuhan, China, 1:500) was then added and incubated for 60 min in the dark. DAPI (Beyotime, Shanghai, China) was added dropwise, and the cells were incubated for 10 min. The cells were evaluated and imaged under fluorescence microscopy (Olympus, Tokyo, Japan).

### 2.5 Cell counting kit-8 (CCK-8) assay

DPCs were seeded into 96-well plates. After 12 h, the medium was replaced with medium containing different concentrations of VE, and a control group (with an equal amount of DMSO) was set up. Due to the broad range of VE tolerance by DPCs, different concentration gradients were used, with the establishment of four treatment groups (25 μM, 50 μM, 100 μM, 200 μM). Next, 10 μL of CCK-8 reagent (Vazyme, Nanjing, China) was added to each well and incubated for 2 h at 37°C. The absorbance at 450 nm was measured using a microplate reader (Chike, Shanghai, China). Measurements were performed at 24 h and 48 h.

### 2.6 RNA extraction, RNA reverse transcription, and reverse-transcription quantitative polymerase chain reaction (RT-qPCR)

RNA from hair follicles and cells was extracted using a SteadyPure RNA extraction kit (Accurate, Hunan, China) and was reverse-transcribed to cDNA using a reverse-transcription kit (Accurate), as shown (Supplementary Table 2). The reverse-transcription PCR program was as follows: 37°C for 15 min and 85°C for 5 s. Specific primers were designed based on the coding regions (CDS) of the genes (Supplementary Table 3).

### 2.7 Western blotting

DPCs were lysed with RIPA lysis buffer (Beyotime). The protein concentration was determined using a bicinchoninic acid kit (Beyotime). Aliquots of total protein were separated on SDS-PAGE and transferred to polyvinylidene fluoride (PVDF) membranes (Beyotime). The membranes were blocked with TBST (Solarbio) containing 5% nonfat milk for 1 h at room temperature (25°C), and subsequently incubated overnight at 4°C with primary antibodies against TIMP1 (Boster, 1:5000), SFRP2 (Boster, 1:5000), STAT1 (Proteintech, 1:10000), and GAPDH (Proteintech, 1:100000) diluted in 5% BSA. The following day, the membrane was washed with TBST and incubated with HRP-conjugated secondary antibody (Proteintech, 1:10000) for 2 h at room temperature. The immunoreactive bands were visualized using an enhanced chemiluminescence detection system (Beyotime), and images were acquired using a chemiluminescence imaging system (Tanno, Shanghai, China).

### 2.8 Determination of antioxidant indicators

The effects of VE on the antioxidant capacity of DPCs were assessed by measuring glutathione peroxidase (GPX, Keming, Suzhou, China) activity, malondialdehyde (MDA, Keming) content, and reactive oxygen species (ROS, Beyotime) levels. Antioxidant capacity was determined by comparing the cell viability of DPCs before and after VE treatment.

### 2.9 Vector construction and synthesis of interference sequences

An overexpression vector was constructed based on the full-length lncRNA2919 sequence (Supplementary Table 4). The target fragment was ligated into the pcDNA3.1^+^ eukaryotic vector. The shRNA lentiviral interference vector was synthesized (GenePharma) (Supplementary Table 4). Primers were designed based on the STAT1 CDS sequence (Supplementary Table 4), and the pcDNA3.1-STAT1 overexpression vector was constructed. The siRNA lentiviral interference vector was constructed (GeneCreate) (Supplementary Table 4).

### 2.10 Cycloheximide (CHX) and MG132 inhibitor treatment

When 70–80% confluent, DPCs were transfected with either pcDNA3.1-lncRNA2919, pcDNA3.1, shRNA-2919, or NC, and the medium was refreshed 6–8 h post-transfection. The protein synthesis inhibitor cycloheximide (CHX) and proteasome inhibitor MG132 (MedChemExpress, Monmouth Junction, NJ, USA) were added 24 h after transfection. For CHX treatment, four cell groups were treated with 30 μg/mL CHX, and proteins were harvested at 0, 2, 4, and 8 h after treatment. For MG132 treatment, four cell groups were treated with 10 μM MG132 for 4.5 h before total protein collection.

### 2.11 Construction of the luciferase reporter gene vector

Primer sequences for p1-SHH, p2-SHH, p3-SHH, and p4-SHH were designed (Supplementary Table 4) to construct dual-luciferase reporter vectors containing different deleted fragments of the *SHH* promoter. The amplified fragment was ligated into the pGL3.0-basic vector. The sequence “CATTCCCAGGATGC” was mutated to “CAATTCCCAGGATGC” using Vazyme online CE Design software (Supplementary Table 4). Subsequently, the SHH mutant plasmid was constructed using the Vazyme Mut Express II Fast Mutagenesis Kit V2 (Vazyme, Nanjing, China).

### 2.12 Dual-luciferase reporter assay

The dual-luciferase reporter assay was performed using a dual-luciferase reporter assay kit (Vazyme). The reporter plasmid and the internal control plasmid (pRL-TK) were transfected into cells in a 10:1 ratio. After 24 h, the medium was discarded. Next, 90 μL of 1× cell lysis buffer was added to each well, and the lysate was centrifuged at 11,800×*g* for 2 min to obtain the supernatant. For the assay, 100 μL of luciferase substrate was placed in each well of an opaque microplate, followed by the addition of 20 μL of the cell supernatant. The contents were mixed, and firefly luciferase activity was measured. Then, 100 μL of Renilla luciferase substrate working solution was added and mixed, and the Renilla luciferase activity was determined.

### 2.13 DNA pull-down assay

Biotin-labeled primers were designed and synthesized based on the full-length sequence of the *SHH* gene promoter region (Supplementary Table 4). The reaction reagents were added in sequence, PCR amplification was performed, and the biotin-labeled DNA product was obtained. The target fragment was recovered and purified to determine the quality of the DNA fragment. Protein extraction, magnetic bead-probe binding, protein incubation, and elution were conducted, followed by Western blotting and analysis using a rapid silver staining kit (GeneCreate, Wuhan, China).

### 2.14 Chromatin immunoprecipitation (ChIP) assay

Formaldehyde (37%) was added to the sample and incubated at 37°C for 10 min to crosslink the protein and DNA. The reaction was terminated with glycine. The cells were then centrifuged at 2000 ×g for 5 min, and the pelleted cells were lysed with cell lysis buffer for 30 min, followed by sonication in an ice bath. After centrifugation at 13,500×*g* and 4°C for 10 min, the supernatant was collected. Protein A/G magnetic beads and salmon sperm DNA were added to the supernatant and incubated at 4°C for 2 h to enrich the complex. The supernatant was discarded, and the beads were washed. Freshly prepared elution buffer was added, followed by NaCl and proteinase K. After de-crosslinking at 55°C overnight, the DNA samples were recovered using a DNA purification and recovery kit (TIANGEN, Beijing, China).

### 2.15 Data processing and analysis

Experimental data were analyzed using SPSS 25.0 and were compared using one-way ANOVA (for multiple groups) or *t*-tests (for two groups). GraphPad Prism 9 was used for graphics. Each experiment was performed with three biological replicates. Results are expressed as mean ± standard deviation. *P* < 0.05 indicates a statistically significant difference, and *P* < 0.01 indicates a highly statistically significant difference.

## 3 Results

### 3.1 Effects of different VE concentrations on hair-follicle growth *in vitro*

**Fig. 1:**
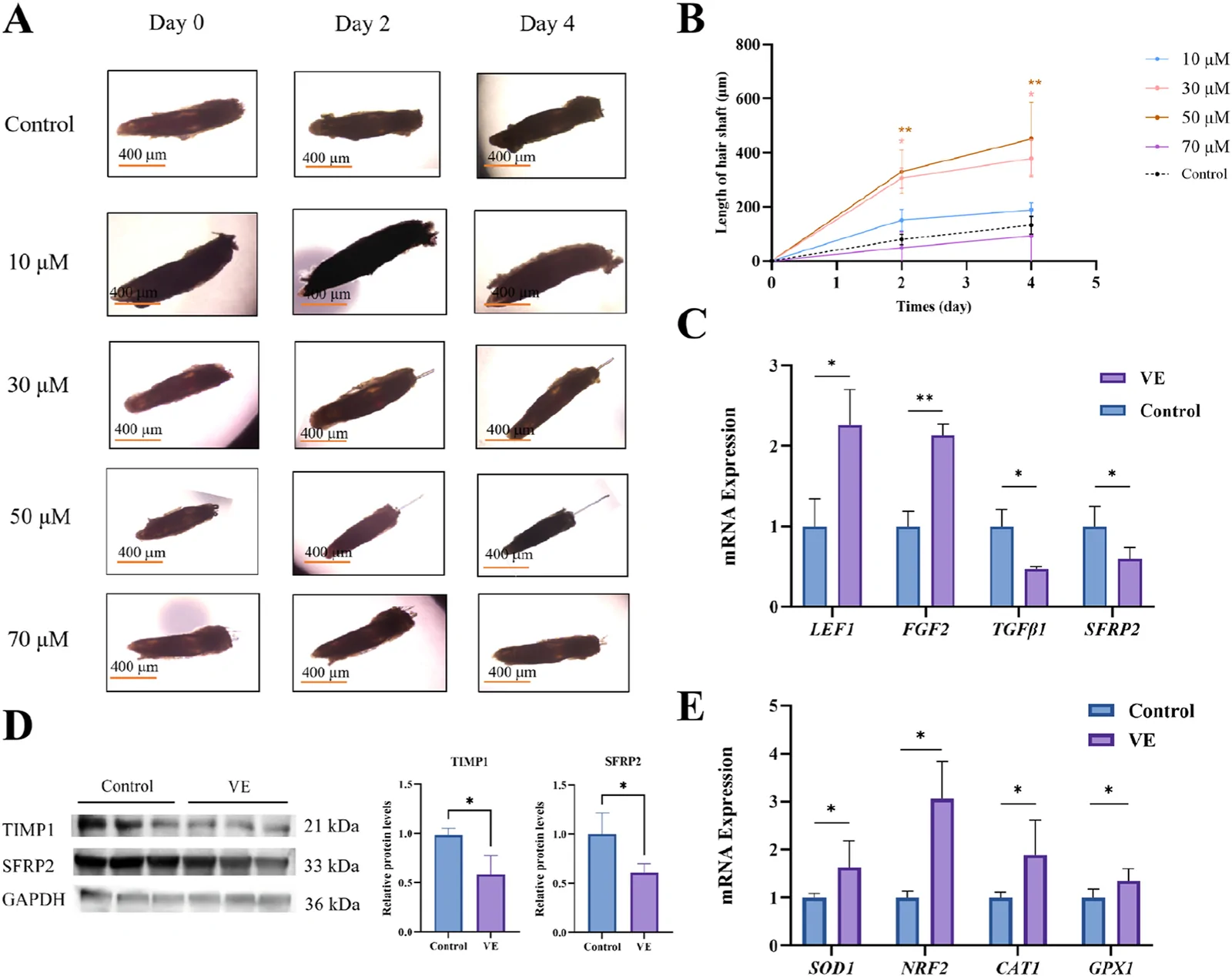
**Effects of VE on hair-follicle growth *in vitro*.** (A) Morphological images of hair shaft growth in isolated hair follicles treated with different VE concentrations. (B) Quantification of hair-shaft growth in isolated hair follicles treated with different VE concentrations. (C) Effects of VE on the mRNA expression of hair follicle development–related genes in isolated hair follicles. (D) Effects of VE on the expression of hair follicle development–related proteins in isolated hair follicles. (E) Effects of VE on the mRNA expression of antioxidant-related genes. Asterisks indicate differences between the treatment and control groups at the same treatment time. *P < 0.05, **P < 0.01.

Different concentrations of VE were co-cultured *in vitro* with individual hair follicles to evaluate the effects on hair-shaft growth in isolated hair follicles. Compared with the control group, growth was observed in the hair follicles in the 30 μM and 50 μM treatment groups, while no significant effects were found in the 10 μM and 70 μM groups (Fig. 1A). Statistical analysis of hair-shaft lengths (Fig. 1B) revealed that 10 μM VE had no significant effect on hair-shaft growth of isolated hair follicles, 30 μM VE significantly promoted hair-shaft growth (*P* < 0.05), and 50 μM VE induced highly significant growth (*P* < 0.01), while growth was inhibited at 70 μM VE.

The effects of VE treatment on the expression of hair follicle development–related genes in isolated hair follicles were determined using RT-qPCR and Western blotting. The expression of *LEF1* and *FGF2* was significantly upregulated (*P* < 0.05), while that of *TGFβ1* and *SFRP2* was significantly decreased (*P* < 0.05) (Fig. 1C). Expression of SFRP2 and TIMP1 proteins was markedly downregulated (*P* < 0.05) (Fig. 1D). Analysis of changes in the expression of antioxidant-related genes indicated that the mRNA levels of *SOD1*, *NRF2*, *CAT1*, and *GPX1* were significantly upregulated after VE treatment compared with those in the control group (*P* < 0.05) (Fig. 1E).

### 3.2 Effects of different concentrations of VE on DPC proliferation

**Fig. 2:**
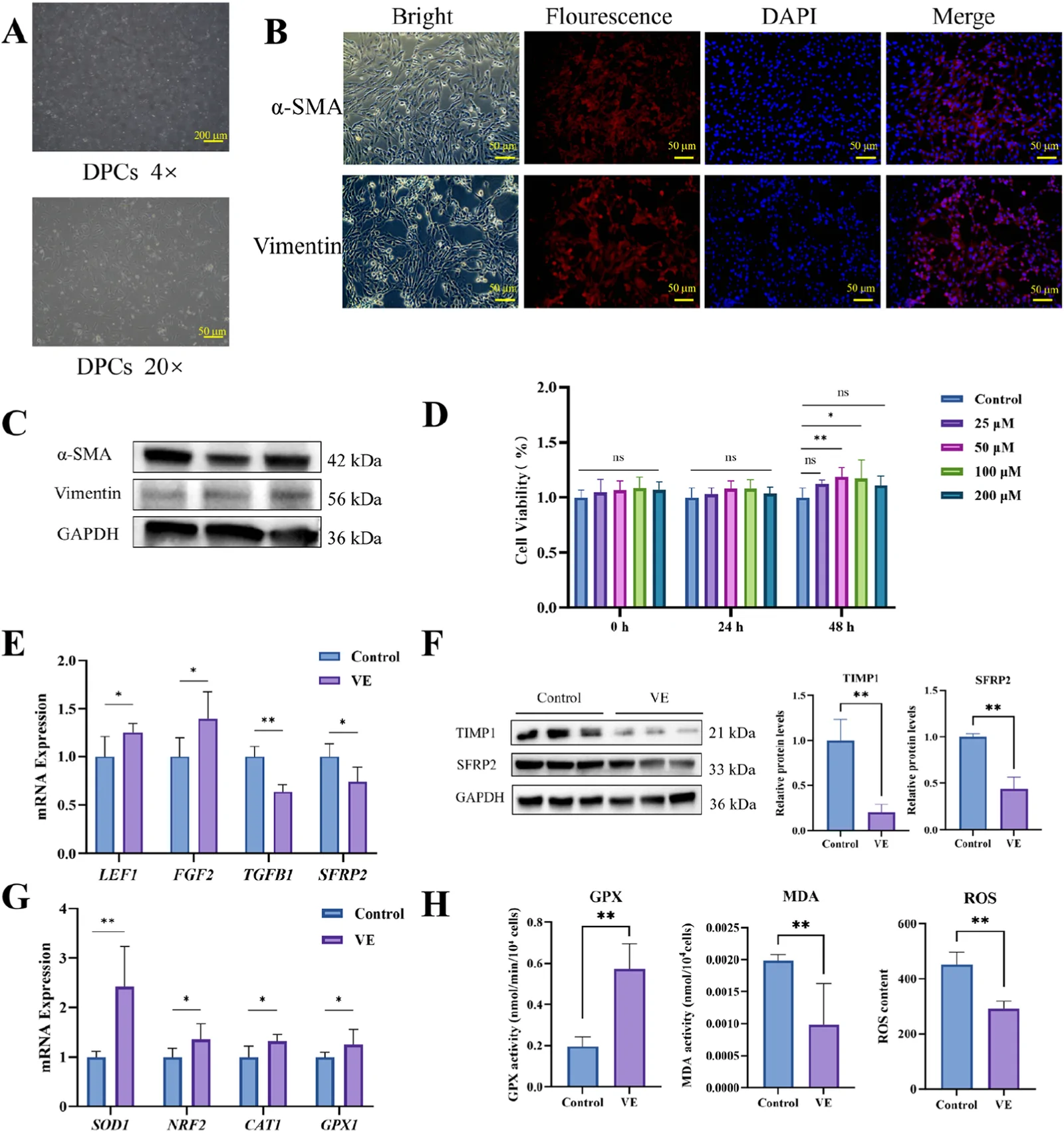
**Effects of VE on the proliferation and antioxidant capacity of DPCs.** (A) DPC growth morphology. (B) Identification of DPCs using immunofluorescence staining for α-SMA and vimentin. (C) Identification of DPCs using Western blotting detection of α-SMA and vimentin proteins. (D) Effects of different concentrations of VE on DPC proliferation. (E) Effects of VE on the mRNA expression of hair follicle development–related genes in DPCs. (F) Effects of VE on the expression of hair follicle development–related proteins in DPCs. (G) Effects of VE on the mRNA expression of antioxidant-related genes. (H) Effects of VE on the antioxidant capacity of DPCs. Asterisks indicate differences between the treatment and control groups at the same treatment time. *P < 0.05, **P < 0.01.

The isolated primary DPCs exhibited long, spindle-shaped or polygonal morphology (Fig. 2A). The results of immunofluorescence staining revealed specific expression of the DPC marker proteins, α-SMA and vimentin (Fig. 2B). Findings from Western blotting indicated distinct bands for α-SMA and vimentin, with the band sizes matching the molecular weights of the target proteins (Fig. 2C). The isolated primary cells were therefore confirmed to be DPCs.

Four concentration gradients for VE (25 μM, 50 μM, 100 μM, 200 μM) were established along with a blank control group. Cell proliferation levels were determined at 0 h, 24 h, and 48 h of culture. There were no significant differences in cell proliferation levels between the control group and the groups treated with different concentrations of VE at 0 h and 24 h of culture. After 48 h of culture, the proliferation capacity of DPCs in all VE-treated groups was significantly higher than that in the control group (*P* < 0.05), with the 50 μM group showing a highly significant increase in DPC proliferation (*P* < 0.01) (Fig. 2D). RT-qPCR analysis revealed significantly upregulated mRNA expression of *LEF1* and *FGF2* (*P* < 0.05) while *TGFβ1* and *SFRP2* were markedly downregulated after VE treatment (*P* < 0.05) (Fig. 2E). Western blotting showed that VE treatment resulted in a highly significant reduction in the protein expression of SFRP2 and TIMP1 (*P* < 0.01) (Fig. 2F).

Further examination of the effects of VE on the antioxidant capacity of DPCs revealed a significant increase in the mRNA expression of *SOD1*, *NRF2*, *CAT1*, and *GPX1* (*P* < 0.05 or *P* < 0.01) (Fig. 2G). Measurement of GPX, MDA, and ROS levels indicated that VE treatment led to a highly significant increase in DPC GPX levels, while those of both MDA and ROS were markedly reduced compared with those in the control group (*P* < 0.01) (Fig. 2H). The findings indicate that VE enhanced the antioxidant capacity of DPCs by regulating antioxidant-related genes.

### 3.3 VE inhibits DPC proliferation by promoting lncRNA2919 expression

**Fig. 3:**
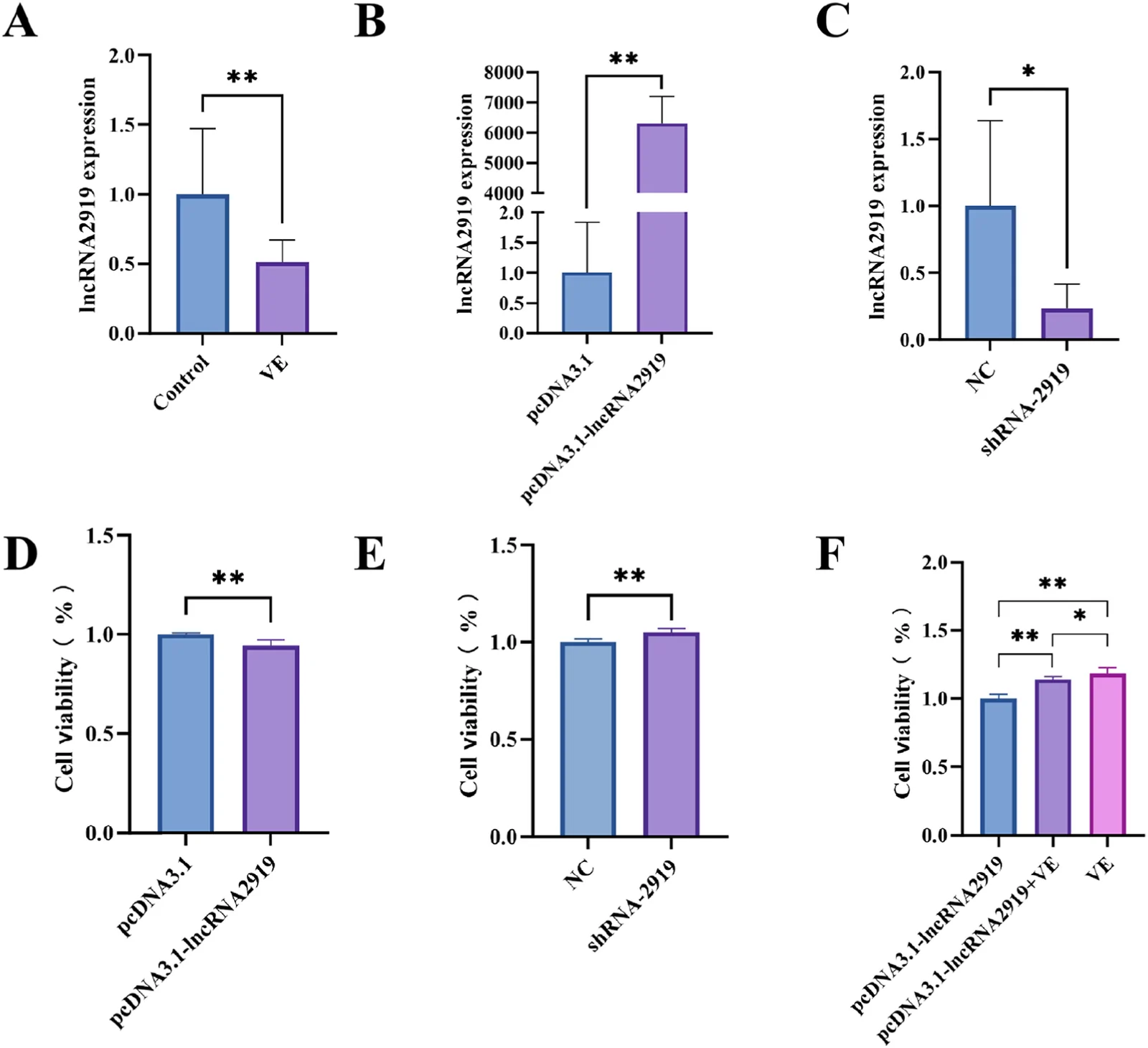
**VE modulates lncRNA2919 levels to promote DPC proliferation.** (A) Effect of VE on lncRNA2919 expression in DPCs. (B) Verification of lncRNA2919 overexpression efficiency. (C) Verification of lncRNA2919 knockdown efficiency. (D) Effect of lncRNA2919 overexpression on DPC proliferation. (E) Effect of lncRNA2919 knockdown on DPC proliferation. (F) Effect of VE on the regulation of DPC proliferation by lncRNA2919. Asterisks indicate between the treatment and control groups at the same treatment time. *P < 0.05, **P < 0.01.

RT-qPCR analysis of VE-treated and untreated DPCs showed that VE led to a highly significant decrease in the expression of lncRNA2919 (*P* < 0.01) (Fig. 3A) compared to the control group, whereas expression was markedly reduced by transfection with shRNA-lncRNA2919 (Fig. 3B and C). CCK-8 assays were used to assess the effects of lncRNA2919 on DPC proliferation, indicating that overexpression of lncRNA2919 led to marked inhibition of the proliferation and viability of DPCs (*P* < 0.01) (Fig. 3D), whereas knockdown of lncRNA2919 enhanced these effects (*P* < 0.01) (Fig. 3E). Rescue experiments were conducted to verify the involvement of VE in regulating lncRNA2919-mediated inhibition of DPC proliferation. The results showed significant recovery of proliferation and viability after lncRNA2919 overexpression and VE treatment group compared with the lncRNA2919 overexpression-only group, indicating that VE could effectively reverse the inhibitory effect of lncRNA2919 on DPC proliferation (*P* < 0.01) (Fig. 3F).

### 3.4 VE modulates lncRNA2919-mediated regulation of STAT1 stability

**Fig. 4:**
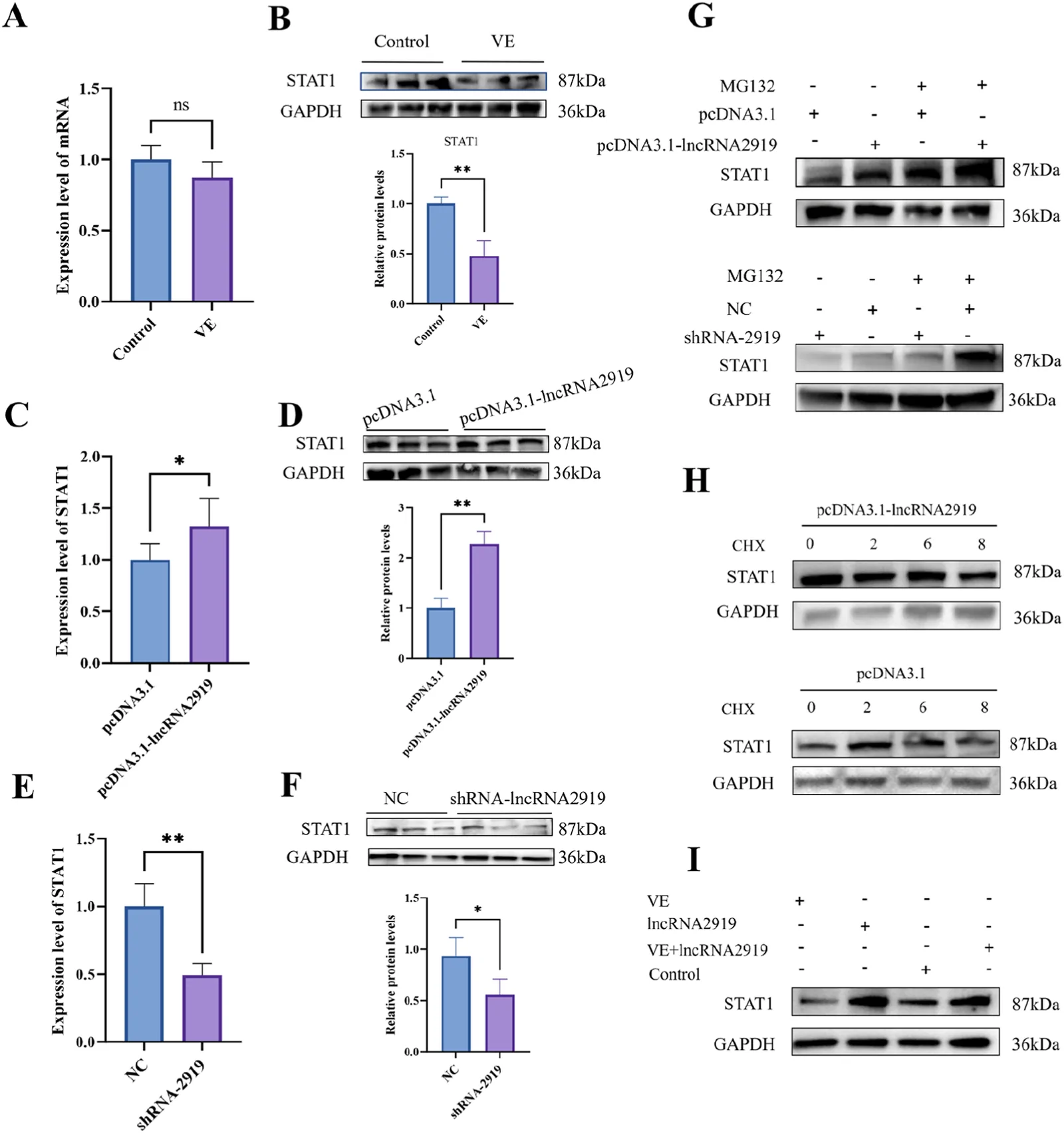
**VE modulates the lncRNA2919/*STAT1* axis.** (A) Effect of VE on *STAT1* transcription. (B) Effect of VE on STAT1 protein expression. (C) Effect of lncRNA2919 overexpression on *STAT1* mRNA expression. (D) Effect of lncRNA2919 overexpression on STAT1 protein expression. (E) Effect of lncRNA2919 knockdown on *STAT*1 mRNA expression. (F) Effect of lncRNA2919 knockdown on STAT1 protein expression. (G) MG132 treatment reveals the regulatory effect of lncRNA2919 on STAT1 protein stability. (H) Effect of lncRNA2919 on STAT1 protein degradation following CHX treatment. (I) Effect of VE on the STAT1 stability mediated by lncRNA2919. Asterisks indicate differences between the treatment and control groups at the same treatment time. *P < 0.05, **P < 0.01.

The mRNA and protein expression of *STAT1* in DPCs was examined after VE treatment. While VE treatment did not significantly affect the transcriptional levels of *STAT1* (*P* > 0.05) (Fig. 4A), the expression of STAT1 protein was significantly downregulated (*P* < 0.05) (Fig. 4B). These findings suggest that VE mainly regulates post-translational expression of STAT1 protein.

Next, the participation of lncRNA2919 in hair-follicle development through regulation of *STAT1* was determined. The RT-qPCR and Western blotting results indicated that lncRNA2919 overexpression significantly upregulated both mRNA and protein expression of *STAT1* (Fig. 4C and D), whereas its knockdown had the opposite effect (*P* < 0.05) (Fig. 4E and F).

MG132 blocks the ubiquitin–proteasome degradation pathway. The characteristics of STAT1 protein accumulation were then examined. Overexpression of lncRNA2919 was observed to significantly increase STAT1 protein levels relative to the controls (Fig. 4G), suggesting that the regulatory effect of lncRNA2919 on *STAT1* is dependent on the proteasomal pathway. To verify the effect of lncRNA2919 on STAT1 protein stability, cellular protein synthesis was blocked using CHX. The results confirmed that lncRNA2919 overexpression significantly inhibited STAT1 protein degradation and prolonged its half-life, thereby enhancing its stability (Fig. 4H).

Western blotting was used to examine the levels of STAT1 in the VE and lncRNA2919 overexpression, lncRNA2919 overexpression, pcDNA3.1 blank control, and VE treatment groups. The results showed that VE treatment significantly reduced *STAT1* expression, in contrast to the upregulated expression observed following lncRNA2919 overexpression. STAT1 levels were reduced compared with those in the lncRNA2919-only treatment group after combined treatment with VE and lncRNA2919 (Fig. 4I). These findings suggest that VE mediates and reverses STAT1 protein upregulation via lncRNA2919, confirming its involvement in the lncRNA2919 regulation of STAT1.

### 3.5 Regulatory role of STAT1 on the transcriptional activity of the *SHH* promoter

**Fig. 5:**
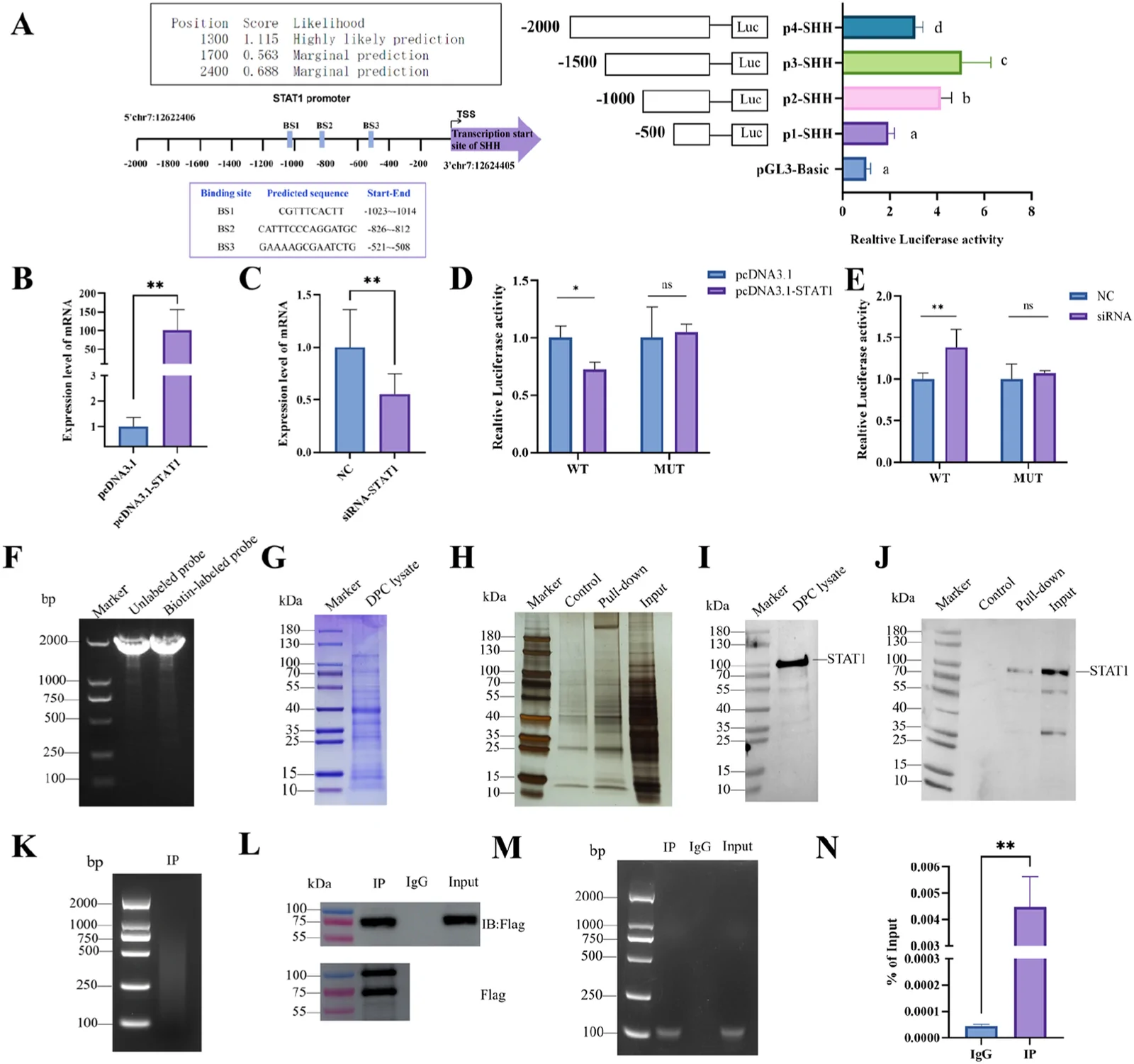
**STAT1 binds to the *SHH* promoter.** (A) Analysis of the transcriptional activity of the *SHH* promoter. (B) Verification of *STAT1* overexpression efficiency. (C) Verification of *STAT1* knockdown efficiency. (D) Effect of *STAT1* overexpression on the transcriptional activity of the *SHH* promoter. (E) Effect of *STAT1* knockdown on the transcriptional activity of the *SHH* promoter. (F) Amplification by the *SHH* promoter probe. Lanes 2 and 3 correspond to the biotin-labeled probe and the unlabeled probe, respectively. (G) Proteins extracted from DPCs. (H) Silver staining of DNA pull-down products. (I) Specific recognition by the anti-STAT1 antibody. (J) Western blotting of the DNA pull-down products. (K) Sonicated chromatin fragments. (L) Immunoblotting of products after ChIP enrichment. (M) Verification of the specificity of the ChIP-qPCR products. (N) Enrichment of STAT1 in the *SHH* promoter region. WT, wild-type *SHH* promoter; MUT, mutant-type *SHH* promoter. Asterisks indicate differences between the treatment and control groups at the same treatment time. *P < 0.05, **P < 0.01.

The Promoter 2.0 online tool was used to predict the core active region of the *SHH* gene promoter. The highest score was predicted for the 1300-bp region of the *SHH* promoter. The dual-luciferase reporter assay findings showed the core transcriptional activity to be between −1500 and −1000 bp, which was consistent with the prediction. Binding elements in the *SHH* promoter were predicted using the JASPAR and Animal TFDB databases, and the *STAT1* binding site was identified in the region from −826 to −812 bp, with the binding sequence “CATTTCCCAGGATGC” (Fig. 5A).

*STAT1* overexpression and knockdown experiments were conducted in DPCs. Confirmation of efficiency indicated significantly higher expression in the *STAT1*-overexpressing cells compared to that in the control group (Fig. 5B), whereas knockdown with siRNA-*STAT1* yielded a significantly lower *STAT1* level than that in the NC group (*P* < 0.01) (Fig. 5C). To investigate the effects of STAT1 on transcriptional activity, DPCs were co-transfected with STAT1 overexpression/knockdown vectors and *SHH*-WT/*SHH*-MUT reporter plasmids. Dual-luciferase assays indicated that STAT1 overexpression significantly inhibited the activity of the *SHH*-WT promoter (*P* < 0.05) (Fig. 5D), whereas *STAT1* knockdown had the opposite effect, with significant enhancement of activity (*P* < 0.01) (Fig. 5E).

The target size of the *SHH* probe was 2000 bp. Both the biotin-labeled and unlabeled probes amplified the target band in this region, indicating that the probe preparation met the quality criteria (Fig. 5F). Proteins were extracted from DPCs, and their quality was verified (Fig. 5G). Silver staining of the DNA pull-down products indicated enrichment of the *SHH* probe in specific binding proteins (Fig. 5H). Western blotting of the extracted proteins was conducted to verify antibody specificity (Fig. 5I). DNA pull-down combined with Western blotting indicated that the transcription factor *STAT1* bound specifically to the *SHH* promoter (Fig. 5G).

The size of the DNA fragments was examined to verify the reliability of the ChIP assay. The DNA fragments in the sample after sonication were mainly between 100 and 500 bp and could be used for subsequent experiments (Fig. 5K). Western blotting of the enriched products was performed to verify the specificity of the immunoprecipitation reaction. STAT1 protein was found to be successfully and specifically enriched (Fig. 5L). ChIP-qPCR was used to assess enrichment of *STAT1* in the promoter region of the *SHH* gene. Compared with that in the IgG group, the DNA enrichment of *STAT1* at the target site of the *SHH* promoter was significantly increased, indicating that *STAT1* bound specifically to the promoter region of the *SHH* gene, suggesting that it promoted the transcription of *SHH* (Fig. 5M and N).

### 3.6 VE modulates lncRNA2919/*STAT1* to regulate the Hedgehog signaling pathway

**Fig. 6:**
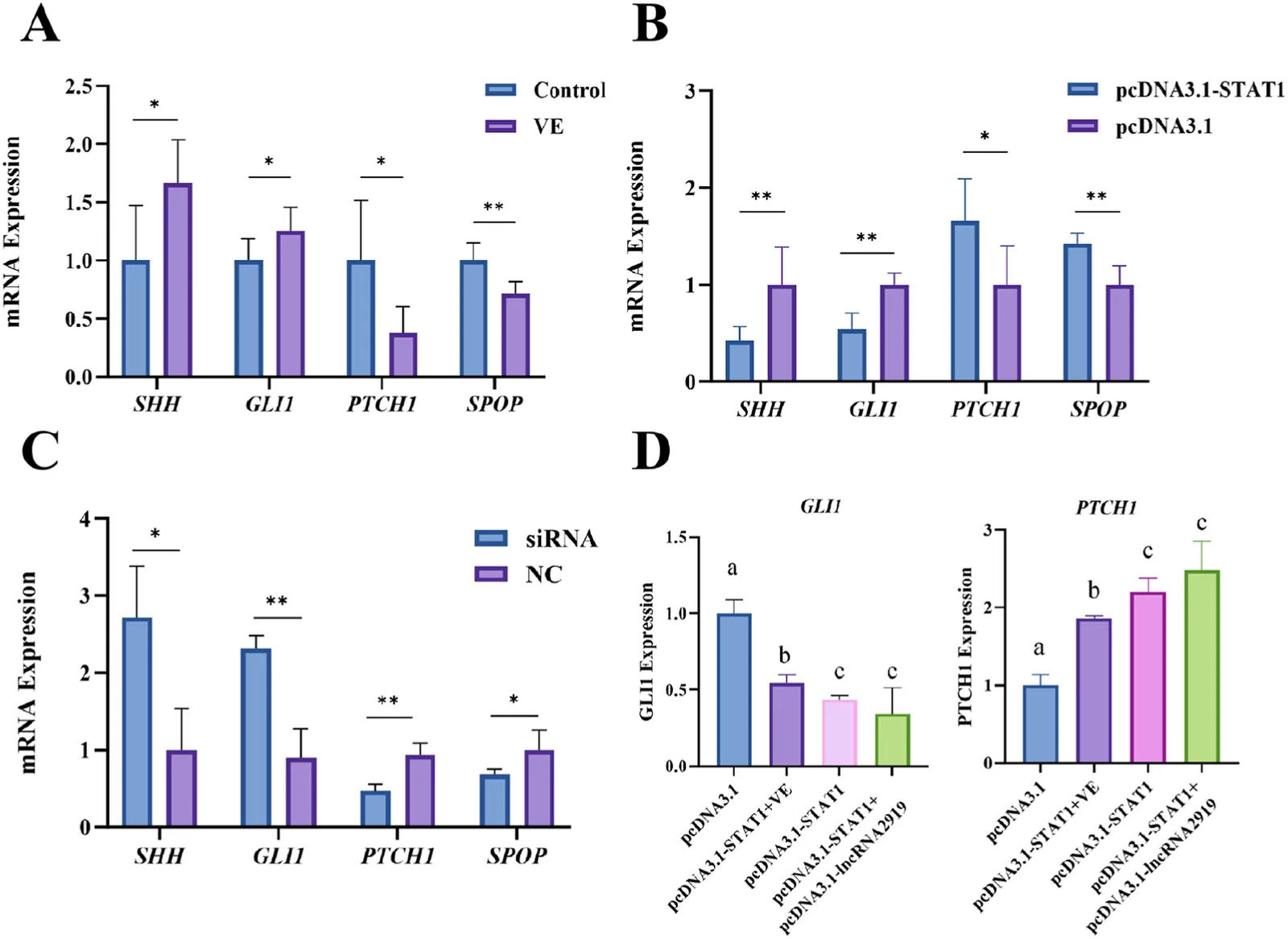
**VE modulates the lncRNA2919/*STAT1* axis to regulate the Hedgehog signaling pathway.** (A) Effects of VE on the mRNA expression of Hedgehog-related genes. (B) Effects of *STAT1* overexpression on Hedgehog signaling pathway–related genes. (C) Effects of *STAT1* knockdown on Hedgehog signaling pathway-related genes. (D) Rescue experiments verifying that VE regulates *GLI1* and *PTCH1* expression through the lncRNA2919/*STAT1* axis. Asterisks indicate differences between the treatment and control groups at the same treatment time. *P < 0.05, **P < 0.01.

To investigate the effect of VE on the Hedgehog signaling pathway, the mRNA expression of key genes in the pathway, *SHH, GLI1, PTCH1,* and *SPOP*, was determined. VE significantly upregulated the expression of *SHH* and *GLI1* and significantly downregulated that of *PTCH1* and *SPOP* (*P* < 0.05), suggesting that it participates in hair-follicle development by regulating the Hedgehog signaling pathway (Fig. 6A).

The regulatory effect of *STAT1* on the Hedgehog signaling pathway was then evaluated. Overexpression of *STAT1* resulted in highly significant downregulation of the mRNA expression of both *SHH* and *GLI1* (*P* < 0.01) and significant upregulation of *PTCH1* and *SPOP* (*P* < 0.05) (Fig. 6B). *STAT1* knockdown significantly upregulated the mRNA expression of *SHH* and *GLI1* (*P* < 0.05), while markedly downregulating that of *PTCH1* and *SPOP* genes (*P* < 0.01) (Fig. 6C).

Rescue experiments revealed that *STAT1* overexpression downregulated *GLI1* and upregulated *PTCH1*. This effect was further enhanced by co-transfection with lncRNA2919. VE treatment significantly reversed these effects, leading to recovery of *GLI1* expression and reduced *PTCH1* expression, confirming that it restored Hedgehog pathway activity by inhibiting the lncRNA2919/*STAT1* axis (Fig. 6D).

**Fig. 7:**
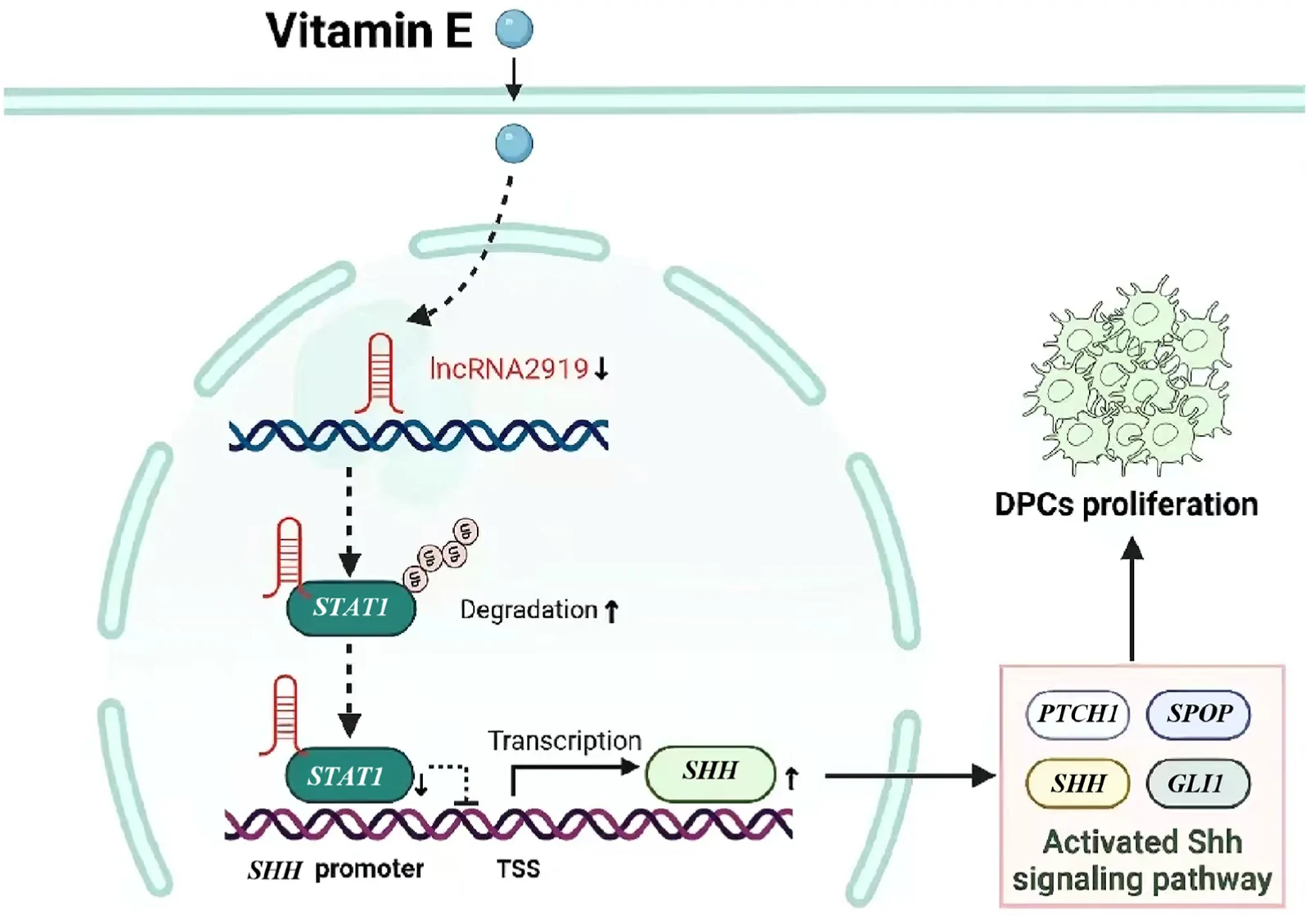
**Schematic diagram of the effects of VE-associated signal transduction on DPC proliferation *via* regulation of the lncRNA2919/*STAT1*/*SHH* axis.** ↑ indicates upregulation,↓ and indicates downregulation. Dashed arrows indicate indirect regulation, and solid arrows indicate direct promotion.

Collectively, the findings demonstrate that VE promotes hair-follicle growth and DPC proliferation *in vitro*. Mechanistically, VE suppresses lncRNA2919 expression, thereby attenuating lncRNA2919-mediated promotion of STAT1 protein stability, thereby reducing the levels of STAT1. As a transcription factor, STAT1 negatively regulates the Hedgehog signaling pathway, thereby participating in hair-follicle growth. Specifically, VE modulates the lncRNA2919/*STAT1* axis to regulate the Hedgehog signaling pathway and promote DPC proliferation, thereby contributing to hair-follicle growth (Fig. 7).

## 4 Discussion

Hair follicles are essential skin appendages that play a critical role in the growth of animal hair and the control of both hair quality and quantity [27]. DPCs are specialized mesenchymal cells that regulate hair-follicle morphogenesis and growth cycle. They are also the core cell type used in research on hair regeneration [28]. The activities of hair-follicle stem cells and hair-cycle transitions depend on DPC-derived signals [29]. VE is a lipophilic antioxidant that interacts directly with lipid radicals, scavenges lipid peroxides, and positively regulates hair-follicle growth and development [30]. In this study, the effects of varying VE concentrations on the hair-follicle tissues and DPCs were analyzed. Treatment with 50 μM VE was found to be optimal for hair-follicle growth and DPC proliferation, and confirmed that VE promoted hair-follicle growth by regulating the expression of hair follicle development–related genes and enhancing the cellular antioxidant capacity.

LncRNAs act as key epigenetic and transcriptional regulators that control hair-follicle morphogenesis, cycle homeostasis, and hair growth [[31], [32], [33], [34], [35]]. Synchronized hair-follicle cycle models have been successfully constructed, and lncRNA2919, identified using whole-transcriptome sequencing, was found to be highly expressed during the catagen phase of the hair follicle [36]. Subsequent functional studies have confirmed that lncRNA2919 is a noncoding nuclear RNA that suppresses DPC proliferation, promotes apoptosis, and inhibits the expression of hair follicle development–related genes [18,26]. In the present study, VE promoted DPC proliferation by inhibiting lncRNA2919 expression, suggesting that lncRNA2919 is a key factor in VE-regulated hair-follicle growth.

LncRNA2919 can recruit *STAT1* to form a transcriptional complex, thereby exerting gene regulatory functions [25]. STAT1 is involved in regulating human DPC survival, hair-cycle transition, and immune repair, among various other activities [19,37]. LncRNAs regulate genes via several mechanisms [[31], [32], [33], [34], [35]], principally by binding directly to target proteins and thereby regulating their translation [38]. LncRNA DRAIC has been shown to bind directly to and inhibit *FBXO11*-mediated ubiquitination and proteasomal degradation and enhance the protein stability of hnRNPA2B1 [39]. LncRNA CCAT1 interacts with PTBP1 to promote the stability of PTBP1 protein by inhibiting its ubiquitin-mediated degradation [40], while lncRNA RMST can enhance SUMO1 modification of FUS protein, promote the interaction between FUS and hnRNPD, and stabilize its expression [41]. Specifically, in the present study, lncRNA2919 was found to directly regulate the stability of STAT1, while VE downregulated lncRNA2919 expression, thereby abolishing the protein-stabilizing effect of lncRNA2919 on STAT1, accelerating STAT1 protein degradation, and achieving the negative regulation of the *STAT1* signaling pathway, ultimately mediating the regulation of hair-follicle growth.

The Hedgehog signaling pathway is an important pathway that regulates the development of the epidermis and hair follicles [42]. Previous studies have confirmed the key role of this pathway in hair-follicle regeneration [43,44]. *SHH* encodes a ligand within the Hedgehog pathway, and is crucial for hair-follicle morphogenesis [45]. In this study, DNA pull-down and ChIP-seq were used to confirm that STAT1 directly binds to the promoter region of the *SHH* gene, thereby inhibiting its transcription. A previous study used a chemotherapy-induced model of alopecia and found that STAT1 binds directly to the *SHH* promoter and that STAT1 deficiency restores *SHH* expression and alleviates hair loss [46]. Furthermore, STAT1 has been found to interact directly with the *SHH* promoter in neural precursor cells to regulate interferon-γ–induced cell proliferation [21]. STAT1 also participates in regulating the Janus kinase*/STAT1* signaling axis and affects the microenvironment of hair-follicle stem cells [19]. The genes in the Hedgehog pathway include *GLI1*, *GLI2*, and *GLI3*, as well as the receptors *PTCH1* and *SMO*, among others. Collectively, they constitute the regulatory network of Hedgehog signal transduction [47]. In the present study, STAT1 overexpression significantly inhibited the expression of *SHH* and *GLI1* and promoted that of the negative regulatory factors *PTCH1* and *SPOP*. STAT1 knockdown exerted the opposite effects. These findings indicate that STAT1 negatively regulates the activity of the Hedgehog signaling pathway by direct inhibition of *SHH* transcription, and also clarify its downstream molecular network.

The findings revealed that VE, by targeting the lncRNA2919/*STAT1* signaling axis, inhibited the expression of STAT1 protein (rather than *STAT1* mRNA) and counteracted its transcriptional inhibition of the *SHH* gene, thereby activating the Hedgehog signaling pathway and promoting DPC proliferation and antioxidant capacity. Collectively, these findings identify a key regulatory mechanism by which VE regulates the lncRNA2919/*STAT1*/Hedgehog signaling network.

## Ethical approval

All animal experimental procedures in this study have been approved by the Animal Care and Use Committee of Yangzhou University (Approval No: 202301018).

## Availability of data and material

The datasets generated and/or analyzed during the current study are available from the corresponding author upon reasonable request.

## Funding

This study was supported by the following project: the 10.13039/501100004608Natural Science Foundation of Jiangsu Province (Grant No. BK20231332) and Zhejiang Key Laboratory of Livestock and Poultry Biotech Breeding.

## CRediT authorship contribution statement

**Yinuo Fang:** Data curation, Formal analysis, Methodology, Writing – original draft. **Sen Wang:** Conceptualization, Investigation, Methodology, Writing – original draft. **Chengpeng Cao:** Conceptualization, Data curation, Formal analysis. **Anqi Yang:** Formal analysis, Investigation. **Jing Zhang:** Formal analysis, Investigation. **Bohao Zhao:** Methodology, Resources, Writing – review & editing. **Xinsheng Wu:** Methodology, Resources, Writing – review & editing. **Yan Liu:** Supervision, Writing – review & editing. **Yang Chen:** Conceptualization, Funding acquisition, Supervision, Writing – review & editing.

## Declaration of competing interest

The authors declare that they have no known competing financial interests or personal relationships that could have appeared to influence the work reported in this paper.
