# Supplementary material for: Vitamin E promotes dermal papilla cell proliferation via the lncRNA2919/STAT1 axis to activate the Hedgehog signaling pathway
Source: Noncoding RNA Res. 2026 Aug 18;20:123–33. doi: 10.1016/j.ncrna.2026.06.005 (PMC13520907; doi:10.1016/j.ncrna.2026.06.005)
Supplement: Supplementary file 1 [file mmc1.docx]

**Appendix A. Supplementary data**

**Supplementary tables**

**Table S1. Preparation of basal culture medium**

| Reagents | Volume |
| --- | --- |
| Hydrocortisone | 0.4 ng/μL |
| L-Glutamine | 0.5 mL |
| Insulin-transferrin-selenium (ITS) solution, 100× | 0.5 mL |
| 1% penicillin-streptomycin solution | 0.5 mL |
| William’s E medium | 50 mL |

**Table S2. Reaction system for RNA reverse transcription**

| Reagent name | 20-μL system |
| --- | --- |
| gDNAClean Reaction Mix | 2 μL |
| 5× Evo M-MLV RT Reaction Mix | 4 μL |
| RNA | 500 ng |
| RNase-free ddH_2_O | to 20 μL |

**Table S3. RT-qPCR primers**

| Gene name | F primer (from 5′ to 3′) | R primer (from 5′ to 3′) |
| --- | --- | --- |
| *GPX1* | TTGGGCATCAGGAGAACGC | TCCAGGCCGGACATACTTGA |
| *CAT1* | ACCGAAACGCTCGGTTACAT | GTGGTCAGGACATCGGGTTT |
| *NRF2* | TGAAAGGTCACTCAGAGCGG | TCTGTGCTGCCATCTTGTCC |
| *SOD1* | GAGTCATGGCGACGAAGGC | CAGTCCTGTTATGCGTCCCT |
| *LEF1* | CATCTCGGGTGGATTCAGG | ATGAGGGATGCCAGTTGTG |
| *FGF2* | GTGTGTGCAAACCGTTACCTT | TCGTTTCAGTGCCACATACCAG |
| *SFRP2* | CCAGCCCGACTTCTCCTACAAGC | TCCAGCACCTCTTTCATGGTCT |
| *TGF-β1* | CAGGTCCTTGCGGAAGTCAA | CTGGAACGGGCTCAACATCTA |
| *STAT1* | GGACGAGGTTCTGCAAGGAA | CTCCTTGCTGATGAAGCCCA |
| *GAPDH* | CACCAGGGCTGCTTTTAACTCT | CTTCCCGTTCTCAGCCTTGACC |
| *SHH* | TGTGGACATCACCACGTCTG | TCTCTGCTTTCACCGAGCAG |
| *PTCH1* | GCCGGGGTTTTTACACTTTCC | TTCCCTGTCGCCGTCTTTTT |
| *CLI1* | GACTGTCTGGTCTGCCCTTC | TAGACAGTGCCCGCTTCTTG |
| *SPOP* | CGGATGTTGCGGACAGTATG | CAGGAAACCTCTGAGAGCGG |

**Table S4. Primer used for the construction of the vector and shRNA sequences**

| Primer name | Primer sequences (from 5′ to 3′) |
| --- | --- |
| pcDNA3.1-IncRNA2919 F | CTAGCTAGCATACTCAAGAAACTCGTCCTTGACG |
| pcDNA3.1-IncRNA2919 R | CCGGAATTCTGTCGTCCCCCACTGCAA |
| ShRNA-IncRNA2919 | GGATCAGGAAGAATGCCTTGC |
| IncRNA2919-NC | GTTCTCCGAACGTGTCACGT |
| p1-*SHH* | cgagctcttacgcgtgctagcGCTTTTATGGGTTCGATGAGATTG |
| p2-*SHH* | cgagctcttacgcgtgctagcATAACAACTTCCAAAGTGAAACGGG |
| p3-*SHH* | cgagctcttacgcgtgctagcGGCTCCAGAAAAGCGAATCTG |
| p4-*SHH* | cgagctcttacgcgtgctagcATGATTTCAAGGTCCTTATAGAGCCC |
| pGL3.0-Basic3-R | cagtaccggaatgccaagcttGATGTGCAAAATCCAGCCGA |
| MUT | F: AAAGTGCAaTTCCCAGGATGCCACTGGTTCAC |
|  | R: CCTGGGAAtTGCACTTTGGTGGGTGCTATTTA |
| pcDNA3.1-*STAT1* | F: GGGAGACCCAAGCTGGCTAGCATGTCTCAGTGGTATGAGCTTTCAGC |
|  | R: TCCGAGCTCGGTACCAAGCTTCTATACTGCGTTCATCATACTGTCGA |
| siRNA-*STAT1* | GGAAGGAAAUCGUUCACAAdTdT |
| *STAT1*-NC | UUCUCCGAACGUGUCACGUTT |
| *SHH* Biotin F | ATGATTTCAAGGTCCTTATAGAG |
| *SHH* Cold F | ATGATTTCAAGGTCCTTATAGAG |
| *SHH* R | GATGTGCAAAATCCAGCCG |
| ChIP-*SHH* | F: TTCTAAATAGCACCCACC |
|  | R: GCCTCAGTCTTACCCATC |
